# Supplementary material for: Resolving the full spectrum of human genome variation using Linked-Reads
Source: Genome Res. 2019 Apr;29(4):635–45. doi: 10.1101/gr.234443.118 (PMC6442396; doi:10.1101/gr.234443.118)
Supplement: Supplemental Material [file supp_gr.234443.118_Supplemental_longranger-2.2.zip › longranger-2.2.tar.bz2!/lr2.2-public/lib/stan/cmdstan/stan/doc/api/html/classstan_1_1memory_1_1stack__alloc.html]

Stan: stan::memory::stack\_alloc Class Reference


|  |  |
| --- | --- |
|  | Stan  2.5.0  probability, sampling & optimization |


- Main Page
- Related Pages
- Namespaces
- Classes
- Files

- Class List
- Class Index
- Class Hierarchy
- Class Members

All Classes Namespaces Files Functions Variables Typedefs Enumerations Enumerator Friends Macros Pages

- stan
- memory
- stack\_alloc

Public Member Functions |
List of all members

stan::memory::stack\_alloc Class Reference

An instance of this class provides a memory pool through which blocks of raw memory may be allocated and then collected simultaneously.
More...

`#include <stack_alloc.hpp>`

|  |  |
| --- | --- |
| Public Member Functions | |
|  | stack\_alloc (size\_t initial\_nbytes=DEFAULT\_INITIAL\_NBYTES) |
|  | Construct a resizable stack allocator initially holding the specified number of bytes. More... |
|  | |
|  | ~stack\_alloc () |
|  | Destroy this memory allocator. More... |
|  | |
| void \* | alloc (size\_t len) |
|  | Return a newly allocated block of memory of the appropriate size managed by the stack allocator. More... |
|  | |
| void | recover\_all () |
|  | Recover all the memory used by the stack allocator. More... |
|  | |
| void | start\_nested () |
|  | Store current positions before doing nested operation so can recover back to start. More... |
|  | |
| void | recover\_nested () |
|  | recover memory back to the last start\_nested call. More... |
|  | |
| void | free\_all () |
|  | Free all memory used by the stack allocator other than the initial block allocation back to the system. More... |
|  | |
| size\_t | bytes\_allocated () |
|  | Return number of bytes allocated to this instance by the heap. More... |
|  | |

## Detailed Description

An instance of this class provides a memory pool through which blocks of raw memory may be allocated and then collected simultaneously.

This class is useful in settings where large numbers of small objects are allocated and then collected all at once. This may include objects whose destructors have no effect.

Memory is allocated on a stack of blocks. Each block allocated is twice as large as the previous one. The memory may be recovered, with the blocks being reused, or all blocks may be freed, resetting the stack of blocks to its original state.

Alignment up to 8 byte boundaries guaranteed for the first malloc, and after that it's up to the caller. On 64-bit architectures, all struct values should be padded to 8-byte boundaries if they contain an 8-byte member or a virtual function.

Definition at line 76 of file stack\_alloc.hpp.

## Constructor & Destructor Documentation

|  |  |  |  |  |  |
| --- | --- | --- | --- | --- | --- |
| stan::memory::stack\_alloc::stack\_alloc | ( | size\_t | *initial\_nbytes* = `DEFAULT_INITIAL_NBYTES` | ) |  |

inline

Construct a resizable stack allocator initially holding the specified number of bytes.

Parameters
:   |  |  |
    | --- | --- |
    | initial\_nbytes | Initial number of bytes for the allocator. Defaults to `(1 << 16) = 64KB` initial bytes. |

Exceptions
:   |  |  |
    | --- | --- |
    | std::runtime\_error | if the underlying malloc is not 8-byte aligned. |

Definition at line 133 of file stack\_alloc.hpp.

|  |  |  |  |  |
| --- | --- | --- | --- | --- |
| stan::memory::stack\_alloc::~stack\_alloc | ( |  | ) |  |

inline

Destroy this memory allocator.

This is implemented as a no-op as there is no destruction required.

Definition at line 149 of file stack\_alloc.hpp.

## Member Function Documentation

|  |  |  |  |  |  |
| --- | --- | --- | --- | --- | --- |
| void\* stan::memory::stack\_alloc::alloc | ( | size\_t | *len* | ) |  |

inline

Return a newly allocated block of memory of the appropriate size managed by the stack allocator.

The allocated pointer will be 8-byte aligned.

This function may call C++'s `malloc()` function, with any exceptions percolated throught this function.

Parameters
:   |  |  |
    | --- | --- |
    | len | Number of bytes to allocate. |

Returns
:   A pointer to the allocated memory.

Definition at line 168 of file stack\_alloc.hpp.

|  |  |  |  |  |
| --- | --- | --- | --- | --- |
| size\_t stan::memory::stack\_alloc::bytes\_allocated | ( |  | ) |  |

inline

Return number of bytes allocated to this instance by the heap.

This is not the same as the number of bytes allocated through calls to memalloc\_. The latter number is not calculatable because space is wasted at the end of blocks if the next alloc request doesn't fit. (Perhaps we could trim down to what is actually used?)

Returns
:   number of bytes allocated to this instance

Definition at line 242 of file stack\_alloc.hpp.

|  |  |  |  |  |
| --- | --- | --- | --- | --- |
| void stan::memory::stack\_alloc::free\_all | ( |  | ) |  |

inline

Free all memory used by the stack allocator other than the initial block allocation back to the system.

Note: the destructor will free all memory.

Definition at line 222 of file stack\_alloc.hpp.

|  |  |  |  |  |
| --- | --- | --- | --- | --- |
| void stan::memory::stack\_alloc::recover\_all | ( |  | ) |  |

inline

Recover all the memory used by the stack allocator.

The stack of memory blocks allocated so far will be available for further allocations. To free memory back to the system, use the function free\_all().

Definition at line 184 of file stack\_alloc.hpp.

|  |  |  |  |  |
| --- | --- | --- | --- | --- |
| void stan::memory::stack\_alloc::recover\_nested | ( |  | ) |  |

inline

recover memory back to the last start\_nested call.

Definition at line 203 of file stack\_alloc.hpp.

|  |  |  |  |  |
| --- | --- | --- | --- | --- |
| void stan::memory::stack\_alloc::start\_nested | ( |  | ) |  |

inline

Store current positions before doing nested operation so can recover back to start.

Definition at line 194 of file stack\_alloc.hpp.

---

The documentation for this class was generated from the following file:

- src/stan/memory/stack\_alloc.hpp


---

[ Stan Home Page ]

*© 2011–2014,
Stan Development Team.*
